# Mental health aspects of binge eating disorder: A cross-sectional mixed-methods study of binge eating disorder experts' perspectives

**DOI:** 10.3389/fpsyt.2022.953203

**Published:** 2022-09-15

**Authors:** Brenna Bray, Chris Bray, Ryan Bradley, Heather Zwickey

**Affiliations:** ^1^Helfgott Research Institute, National University of Natural Medicine, Portland, OR, United States; ^2^Wilder Research Division, Amherst H. Wilder Foundation, Saint Paul, MN, United States; ^3^Herbert Wertheim School of Public Health, University of California, San Diego, San Diego, CA, United States

**Keywords:** binge eating, binge eating disorder (BED), eating disorder, mental health, depression, anxiety, attention deficit disorder

## Abstract

Binge eating disorder has high comorbidity with a variety of mental health diagnoses and significantly impairs quality of life. This mixed-methods cross-sectional survey study aimed to collect information from experts in the field about mental health issues pertaining to adult binge eating disorder pathology. Fourteen expert binge eating disorder researchers and clinicians were identified based on history of NIH R01 funding, relevant PubMed-indexed publications, active practice in the field, leadership in related professional societies, and/or distinction in popular press. Semi-structured interviews were anonymously recorded and analyzed by ≥2 investigators using reflexive thematic analysis and quantification. The domains of depression, anxiety, attention deficit disorder (ADD)/attention deficit hyperactive disorder (ADHD), substance-related and addictive disorders (SRADs), obsessive-compulsive disorder (OCD), and post-traumatic stress disorder (PTSD) were addressed in relation to binge eating disorder pathology by 100, 100, 93, 79, 71, and 64% of participants, respectively. Depression and anxiety seem to be the most commonly recognized mental health comorbidities among experts participating in this study. These expert perceptions generally align with the most comprehensive and up-to-date information available on mental health comorbidity prevalence data in adult binge eating disorder, though updated surveys are warranted. The findings from this study highlight the importance of screening for binge eating disorder among individuals with Axis-I mental health diagnoses (e.g., depression and other mood disorders, anxiety disorders, ADD/ADHD, and SRADs). Research on underlying mechanisms that link various Axis-I disorders to binge eating disorder is also warranted and recommended by the experts.

## Introduction

Binge eating disorder is a DSM-5 diagnosis characterized by discrete rapid consumption of objectively large amounts of food due to loss of control associated with marked distress (1). Binge episodes often occur in isolation due to embarrassment and are accompanied by feelings of disgust, depression, and guilt (1). The disorder is associated with high lifetime prevalence rates (4.5–31%) and a complicated health sequelae that includes high comorbidity with a variety of mental health disorders and significantly impaired quality of life (2–14).

The most comprehensive information available on binge eating disorder mental health comorbidities comes from the World Health Organization (WHO) World Mental Health (WHO-WMH) Survey Initiative conducted in 2001–2003 in 24,124 respondents in 30 countries, including 1,929 adults with binge eating disorder (Table 1) (4). The WHO National Comorbidity Replication survey (WHO-NCR) was subsequently conducted through face-to-face interviews administered to a sample of 9,282 adults in the U.S (260 with binge eating disorder) (2). This was followed by a nationally representative study of 35,709 adults with binge eating disorder conducted in the U.S. between 2012 and 2013 (conducted as part of the national epidemiologic survey on alcohol and related conditions III, NESARC-III) (15). In Sweden, a National study was also conducted that assessed psychiatric comorbidities through medical records of 526 adults presenting to specialist clinics for binge eating disorder (records extracted from a large clinical database between 2008 and 2012) (the Stepwise quality control database study, SQCDS) (13). Another Swedish study linked extensive longitudinal data from the Swedish national eating disorders quality registers and patient registers (SNEDQRPR) to assess psychiatric comorbidity in 850 adults with binge eating disorder, with data extracted in 2009 (the SNEDQRPR study, SNEDQRPRS) (14).

**Table 1:** Participant recognition of mental health disorders comorbid with BED (as identified in this study) in comparison to lifetime prevalence rates of various mental health disorders found in sample populations of current global and national prevalence studies.

**Mental health disorder**	***N* (%) participants who described a relationship with BED in this study (*n* = 14 BED experts)**	**WHO-WMH study (4)** **(*n* = 1,929 individuals with BED)**	**WHO-NCR (2)** **(*n* = 260 individuals with BED)**	**U.S. NESARC-III study (15)** **(*n* = 35,709 individuals with BED)**	**SQCDS study (13)** **(*n* = 526 individuals with BED)**	**SNEDQRPR study (14)** **(*n* = 850 individuals with BED)**
Any psychiatric disorder/DSM diagnosis^*^	N/A	4.3%^a^	78.9%	93.8%	74.1%	N/A
Any Axis-I mood disorder^*^	N/A	5.2%^a^	46.4%	69.9%	45.1%	N/A
Depression/major depressive disorder^*^	Comorbid: 13 (93%)	5.1%^$^	32.3%	65.5%	32.3%	23.9%
Any Axis-I anxiety disorder^*^	N/A	5.0%^a^	65.1%^d^	59.0%	55.5%	17.2%^g^
Anxiety/generalized anxiety disorder^*^	Comorbid: 11 (79%)	6.1%	11.8%	33.0%	26.4%	N/A
OCD^**^	Comorbid: 7 (64%)	N/A	8.2%^e^	N/A	1.5%	1.2%
ADD/ADHD^*^	Relevant: 13 (93%) Comorbid: 6 (46%)	9.3%^b^	19.8%^f^	N/A	N/A	1.7%
Any Axis-I SRAD/SUD^*^	Comorbid: 9 (64%)	4.5%^a^	23.3%	67.7%	9.1%	N/A
Alcohol ud^*^	N/A	4.4%	21.4%	52.0%	7.6%	3.2%
Nicotine ud^*^	N/A	N/A	N/A	40.2%	N/A	N/A
Illicit drug ud^*^	N/A	7.6%^c^	19.4%	24.7%	2.9%	2.1%
Any personality or conduct disorder^*^	N/A	N/A	N/A	56%	N/A	N/A
Post-traumatic stress disorder (PTSD)^*^	Comorbid: 6 (46%)	N/A	26.3%	31.6%	N/A	9.4%
Schizophrenia or schizoaffective disorder^*^	N/A	N/A	N/A	N/A	N/A	0.2%
Autism spectrum disorder^*^	N/A	N/A	N/A	N/A	N/A	0.2%

For each study, the most prevalent comorbidity is indicated in bold. The standard errors and confidence intervals for these data are in the original manuscripts.

* Indicates DSM-IV/CIDI disorder.

** At the time of the WHO WMH survey initiative, OCD was classified as an anxiety disorder, but was reclassified as no longer being an anxiety disorder in the DSM-5, used at the time this study was conducted.

$ Describes statistics for major depressive episode/dysthymia^*^.

a Disorders were coded as absent: (1) for countries that did not assess for these disorders, or (2) among respondents who were not assessed for these disorders (*n* = 24,124) (4).

b Excluded New Zealand since it was not assessed for this disorder. Except for Brazil, Romania, and Northern Ireland, all other countries only assessed those who are ≦44 years old for this disorder (*n* = 12,413) (4).

c Excluded Belgium, France, Germany, Italy, Netherlands, Portugal, and Spain, since these countries were not assessed for this disorder (*n* = 19,476) (4).

d OCD was coded as absent among respondents who were not assessed for this disorder (2).

e Restricted to a random sub-sample of respondents (*n* = 1,139) (2).

f Restricted to respondents in the age range 18–44 (*n* = 1,672) (2).

g Any anxiety disorder does not include OCD or PTSD (14).

ud, use disorder; ADD/ADHD, attention deficit disorder/attention deficit hyperactive disorder; BED, binge eating disorder; OCD, obsessive compulsive disorder; PTSD, post-traumatic stress disorder; SRADs, substance-related and addictive disorders; SUD, substance use disorder; WHO, World Health Organization, WMH, World Mental Health surveys (4).

These data have generally found the most prevalent mental health comorbidities in adult binge eating disorder to be depression/any axis-I mood disorder (with sample prevalence rates of 23.9–69.9%) (14, 15) or any axis-I anxiety disorder (sample prevalence rates of 55.5–65.1%) (2, 13) (Table 1). However, the WHO-WMH survey found the highest association between lifetime binge eating disorder and lifetime attention deficit disorder/attention deficit hyperactive disorder (ADD/ADHD) (9.3%^2^, SE = 2.7), with the caveat the data excluded New Zealand (since it was not assessed for ADD/ADHD) and several other countries that only assessed those who were ≦44 years old for ADD/ADHD (4).

Data from these surveys were collected or extracted prior to the release of the *Diagnostic and Statistical Manual of Mental Disorders, 5th Edition* (DSM-5) in 2013, when binge eating disorder became its own autonomous eating disorder diagnosis (1). Previously, binge eating disorder was classified in the DSM-4 as eating disorder not otherwise specified (ED-NOS) (16, 17). The DSM-5 also reclassified OCD as no longer being an anxiety disorder, along with other reconfigurations (1, 17). It should be noted there is not complete agreement in the psychiatric community about the several new diagnoses proposed in the manual. Furthermore, these studies assess individuals who already have a DSM diagnosis of binge eating disorder (and the Swedish studies further assess only treatment-seeking populations with a formal DSM diagnosis). Studies conducted between 2012 and 2015 have found that 93.4–96.8% of individuals who meet DSM criteria for binge eating disorder never receive a formal diagnosis (18, 19); 67.3% do not perceive the need for treatment (18); and 56.4–86.8% never receive or pursue treatment (2, 18). Thus, the true population of adults with binge eating disorder may not be adequately represented by the populations sampled in these surveys. Updated information on mental health correlates of binge eating disorder is warranted.

Here, we conducted and recorded semi-structured interviews with 14 expert binge eating disorder researchers, clinicians, and healthcare administrators and used reflexive thematic analysis to assess collect information from experts in the field regarding whether and how depression, anxiety, attention deficit disorder (ADD)/attention deficit hyperactive disorder (ADHD), substance-related and addictive disorder (SRAD), obsessive-compulsive disorder (OCD), and post-traumatic stress disorder (PTSD) relate to adult binge eating disorder pathology.

## Methods

### Participants and recruitment

Expert researchers, clinicians, and healthcare administrators in the field of adult binge eating disorder were recruited based on the eligibility criteria shown in Table 2.

**Table 2:** Participant eligibility criteria.

**I. Eligibility criteria for researchers (18 recruited, 7 enrolled)**
Researcher eligibility required meeting one of the following criteria:
1. ≥1 active R01 grant on binge eating or food addiction reported on NIH RePORTER (https://report.nih.gov)
2. Last author of ≥10 PubMed**-**indexed publications published in 2010–20 AND ≥5 PubMed-indexed publications in 2015–20 on adult binge eating disorder
3. Last author of ≥5 PubMed publications published in 2015–20 on food addiction^a^
4. Referral from someone meeting one of the qualifications above
**II. Eligibility criteria for clinicians and healthcare administrators (18 recruited, 6 enrolled)**
Clinical eligibility required meeting ≥ 3 of the following criteria:
1. Award Winner or Honoree of the Association of Eating Disorders (AED) (2010–2020) or Castle Connolly Top Doctors Distinction in Psychiatry – Eating Disorders (2020/21) (20, 21)
2. Executive position/board member for one of ten relevant societies: Academy of Nutrition and Dietetics, Academy of Eating Disorders (AED, FAED) (22), American Society for Metabolic and Bariatric Surgery (ASMBS), Behavioral Health Nutrition Society, Eating Disorder Research Society (EDRS), International Association of Eating Disorder Providers (IAEDP), Johns Hopkins 2020 Eating Disorders Conference, National Center of Excellence for Eating Disorders (NCEED), National Eating Disorder Association (NEDA), Obesity Society
3. Adult binge eating disorder provider listed in the Provider Directory for the National Eating Disorder Association (NEDA) (23) or Alliance for Eating Disorders Awareness (24) or associated with an eating disorder program or treatment center with five or more locations listed in the NEDA Directory (23)
4. Popular press recognition in a 2016 New York Times article (25) or a 2021 self-help website for binge eating disorder books (26)
5. Referral from someone meeting ≥ 2 qualifications above
6. Registered Dietician (RD) meeting 2 other criteria above
**III. Additional Eligibility Criteria (2 recruited, 2 enrolled^b^)**
Individuals meeting ≥ 1 research criterion (I.1–4) and ≥1 clinical criterion (II.1–6) were also eligible.

Results expressed as *n* (%). Percentages expressed as *n*/14 times 100.

a This criterion required ≥ five publications in the past 5 years because the concept of food addiction is still relatively new.

b Both participants each met two academic/research criteria and two clinician/healthcare administrator criteria (20–26) refer to citations in References. This table is adapted from that printed in Bray et al. (27), with permission from the authors and editors.

NIH, National Institute of Health.

### Procedure

The procedure was approved by the National University of Natural Medicine (NUNM) IRB (# HZ12120) and is described in Bray et al. (27). BB sent eligible participants a scripted study invitation email. Consenting respondents were anonymously interviewed on Zoom (Zoom.com) using a semi-structured interview script that solicited participants to address their perspectives on how depression, anxiety, ADD/ADHD, SRADs, and OCD relate to adult binge eating disorder.

### Data analysis

Interview recordings were transcribed and de-identified then separately reviewed, qualitatively analyzed, and coded for themes by BB and HZ using reflexive thematic analysis (28). Themes were then discussed and finalized through reflexive engagement with the data (28). BB also quantified the number of participants expressing positive/supportive, negative/skeptical, or neutral perspectives on each identified theme. HZ and CB were consulted when quantitative analysis questions arose and for tiebreakers.

## Results

### Participant response rates and characteristics

Fourteen experts consented, enrolled, and participated in the study (Table 2). Table 3 shows characteristics for the 13/14 participants who provided demographic information.

**Table 3:** Characteristics of the 13/14 study participants who provided demographic data.

**Accreditations**	
Fellow of the Academy of Eating Disorders (FAED)	8 (62%)
Doctor of Philosophy or Science (PhD/ScD)	8 (62%)
Medical Doctor (MD)	4 (31%)
Licensed or Registered Dietician (LD/RD)	4 (31%)
Certified Chef	1 (8%)
Certified Intuitive Eating Specialist (CIES)	1 (8%)
Fellow of the American College of Neuropsychopharmacology (FACNP)	1 (8%)
Bachelor of Medicine Chirurgical Doctor (BMBChB)	1 (8%)
Masters in Public Health (MPH)	1 (8%)
**Sex assigned at birth**	
Female	8 (62%)
Male	5 (38%)
Other	0 (0%)
**Age**	
55 ± 10.2 years (range: 37–44 yrs., *n* = 13)	
**Ethnicity**	
Not Hispanic or Latino	13 (100%)
Hispanic or Latino	0 (0%)
**Race**	
White	12 (92%)
Asian	1 (8%)
American Indian or Alaska Native	0 (0%)
Black or African American	0 (0%)
Native Hawaiian or Other Pacific Islander	0 (0%)
More than one race	0 (0%)
**Geographical location of residence**	**7 reported**
United States of America (USA)	5 (71%)^**^
United Kingdom (UK)	1 (14%)^**^
Australia (AU)	1 (14%)^**^
Canada (CA)	1 (14%)^**^

Results expressed as *n* (%) or mean ± SD.

** Percentages are expressed as *n*/7 times 100, as only seven participants provided this data. This table is adapted from that printed in Bray et al. (27), with permission from the authors and editors.

### Domain 1: Depression

#### Theme 1: Nature of relationship between depression and binge eating disorder

All 14 participants described their views on how depression relates to binge eating disorder pathology (14/14, 100%, Table 4). Thirteen participants (13/14, 93%) described depression as being a common comorbidity with binge eating disorder; two of these participants referenced empirical support. Two participants expressed views that binge eating disorder increases risk for depression (2/14, 14%). One participant expressed a view that depression is associated with increased binge eating disorder symptom severity (1/14, 7%). One participant referenced empirical support that depression is not predictive of binge eating disorder (1/14, 7%). One participant also described depression as possibly masking binge eating disorder, citing that an individual with binge eating disorder may be more likely to seek treatment for their depression than for their binge eating (1/14, 7%). Select quotes from participants regarding the relationship between depression and binge eating disorder are shown below and in Table 4.

“*[Depression and anxiety have] …very high rates of comorbidity with binge eating disorder. …probably the [highest among other comorbidities] … that's true also for substance use disorders, but I think… the evidence seems to suggest that either depression or anxiety has the highest levels of comorbidity, and then after that, maybe substance abuse.” (P38)*.

“*You see a lot of people with [binge eating disorder who also have] depression that's not really treated [or who also have other] under-treated, co-occurring psychiatric illnesses or unaddressed co-occurring [psychiatric illnesses]. [These] medical conditions …can cause fatigue, loss of energy, loss of concentration… and so any other medical issues [that co-occur with binge eating disorder can add to the burden of the disorder itself]. I mean, just bingeing itself is exhausting.” (P7)*

“*Many folks are going to be more open to seeking treatment for obesity and/or even depression/anxiety but not [binge eating disorder]. This might result in the [binge eating disorder] going undiagnosed and untreated for extended periods of time (which has bearing on prognosis).” (P75)*

**Table 4:** Participant statements relating domain 1, “depression” to binge eating disorder pathology (100%).

**Theme 1) Nature of relationship between depression and BED**	**14 (100%)**
1) Common comorbidity between depression and BED	13 (93%)
Referenced empirical support	2 (14%)
2) BED increases risk for depression	2 (14%)
3) Depression associated with increased BED symptom severity	1 (7%)
4) Depression is not predictive of BED^a^	1 (7%)
5) Depression masking BED^b^	1 (7%)
**Theme 2) Possible mechanisms linking depression and BED**	**11 (79%)**
1) Binge eating to cope with depression or regulate stress or mood	6 (43%)
Binge eating to cope	2 (14%)
Binge eating to regulate stress or mood	2 (14%)
Depression increasing one's allostatic stress load/burden^c^	2 (14%)
2) Micronutrient deficiencies in BED can cause depression	4 (29%)
3) Body weight/shape overvaluation linked to depression	3 (21%)
4) Any additional diagnosis can increase distress and thus likelihood of seeking treatment	2 (14%)
5) Consumption of specific foods may link depression and BED	2 (14%)
Consumption of specific foods that can cause inflammatory processes associated with depression	1 (7%)
Consuming a high carbohydrate load can reduce noradrenergic tone (and thus contribute to depression)	1 (7%)
6) Links between socioeconomic status, depression, and BED	2 (14%)
7) Depression increasing sensitivity to weight stigma	1 (7%)
8) Depression increasing appetite	1 (7%)
9) Depression draining energy	1 (7%)
**Additional participant statements related to theme 1, “nature of the relationship”**	
“*I think that the simple comorbidity of [depression] with binge eating is noteworthy. I don't know the direction of the association. I don't know what causes what, I don't know if a third factor causes both.” (P33)*	
“*There's no question that folks with binge eating disorder are at increased risk for depression.” (P46)*	
“*Overall, we know that [binge eating disorder] is highly comorbid with [depression, anxiety, ADD/ADHD, substance-related and addiction disorders, and obesity]. This sometimes creates challenges in determining an accurate [binge eating disorder] diagnosis as many folks may be more likely to present for treatment of their comorbid conditions before presenting for [binge eating disorder] treatment. This is a particular challenge given that these disorders often work in tandem so only addressing one of them can make it harder to achieve recovery.” (P75)*	
**Additional participant statements related to theme 2, “possible underlying mechanisms”**	
“*There is without question, a group, where, you know, if they're **under eating**, [similar to what was observed in the Keys et al. (31) starvation] study, that really **elicits depression** for them.” (P72)*	

Results expressed as n (%). Percentages: *n*/14 times 100.

a Referenced empirical support.

b e.g., an individual with BED may be more likely to seek treatment for their anxiety than for their BED.

c e.g., adding “one more thing to deal with.”

ADD, attention deficit disorder; ADHD, attention deficit hyperactive disorder; BED, binge eating disorder.

#### Theme 2: Possible mechanisms linking depression to binge eating disorder

Eleven participants (11/14, 79%) described possible mechanisms that underlie a relationship between depression and binge eating disorder (Table 4). These included: (1) binge eating to cope with depression or regulate stress or mood (6/14, 43%); (2) micronutrient deficiencies that can occur in binge eating disorder and cause depression (4/14, 29%); (3) body weight/shape/size overvaluation in binge eating disorder contributing to depression (3/14, 21%); (4) any additional diagnosis increasing distress and thus likelihood of treatment-seeking (2/14, 14%); (5) consumption of specific foods linking depression and binge eating disorder (e.g., consumption of processed foods that can cause inflammatory processes associated with depression; high carbohydrate load consumption can reduce noradrenergic tone) (2/14, 14%); (6) links between socioeconomic status, depression, and binge eating disorder (2/14, 14%); (7) depression increasing sensitivity to weight stigma (1/14, 7%); (8) depression increasing appetite (1/14, 7%); and (9) depression draining energy (1/14, 7%). Select quotes from participants regarding possible mechanisms linking depression and binge eating disorder are shown below and in Table 4.

“*I use micronutrient testing to show …all the different nutrients that can impact depression [and] anxiety. …So, we'll look and see if they have deficiencies and those [micronutrients], which oftentimes they do. But I think also their binge eating …is a way of coping with depression and anxiety.” (P37)*

“*If somebody … has a ton of nutritional chaos, they're very likely to be struggling with depression, because their brain is just not getting fed well, and so we have to replete and stabilize before we know if it's really depression, or if it's sort of nutrition-chaos-induced depression.” (P53)*

“*The more diagnoses that people have, the more likely they are to make their way into treatment, and we see the reflection of that for sure.” (P5)*

### Domain 2: Anxiety

#### Theme 1: Nature of relationship between anxiety and binge eating disorder

All 14 participants described their views on how anxiety relates to binge eating disorder pathology (14/14, 100%, Table 5). Thirteen participants (13/14, 93%) described their perspectives on the *nature* of the relationship between anxiety and binge eating disorder. Eleven of these participants described anxiety as being a common comorbidity with binge eating disorder (11/14, 79%). Three of these participants cited research (3/14, 21%). Two estimated anxiety prevalence in binge eating disorder to be similar to that observed in other eating disorders (2/14, 14%); whereas one participant expressed a view that anxiety is less prevalent in binge eating disorder than in other eating disorders (1/14, 7%). One participant cited research that anxiety is “less important” than depression (though still relevant) as it pertains to binge eating disorder (1/14, 7%).

**Table 5:** Participant statements relating to domain 2, “anxiety” (100%).

**Theme 1) Nature of relationship between anxiety and BED**	**13 (93%)**
1) Comorbidity between anxiety and BED	11 (79%)
Common comorbidity between anxiety and BED	11 (79%)
Referenced empirical support	3 (21%)
Anxiety prevalence in BED is similar to that in other EDs	2 (14%)
Anxiety is less prevalent in BED (vs. other EDs)	1 (7%)
Anxiety is “less important” than depression^b^	1 (7%)
2) Anxiety makes BED symptoms or diagnosis more difficult to manage	5 (36%)
3) Anxiety contributes to distress that causes an individual to seek treatment or for the behavior to become diagnostic	2 (14%)
4) Anxiety driven by- or consequence of - BED	2 (14%)
5) Anxiety is not predictive of BED^a^	1 (7%)
6) Anxiety makes it more difficult for patients to maintain engagement with treatment	1 (7%)
7) Anxiety masking BED^b^	1 (7%)
**Theme 2) Possible mechanisms linking anxiety to BED:**	**4 (29%)**
1) Anxiety related to possible food- or micronutrient deficiencies in BED	2 (14%)
2) Anxiety increased by weight gain, fears of gaining weight, being around certain foods, or related to interpersonal experiences	1 (7%)
3) Anxiety related to serotonin dysregulation that occurs in BED	1 (7%)
4) Binge eating to cope with- or soothe anxiety	1 (7%)
**Theme 4) Social anxiety**	**3 (21%)**
1) Referenced research relating social threat to BED	2 (14%)
2) Most common additional mental health problem among EDs	1 (7%)
**Additional participant statements related to theme 1, “nature of the relationship”**	
“*We … tend to associate [anxiety] with anorexia nervosa, and I just think we see it across other types of eating disorders too.” (P72)*	

Results expressed as *n* (%). Percentages: *n*/14 times 100.

a Referenced empirical support.

b As it pertains to BED (though still perceived as relevant).

BED, binge eating disorder; ED, eating disorder.

Five participants described anxiety as making binge eating disorder or its symptoms more difficult to manage (5/14, 36%). Two participants also described anxiety as contributing to the distress that causes an individual to seek treatment or for the behavior to become diagnostic (2/14, 14%). Two participants described anxiety as being driven by—or a consequence of—binge eating disorder (2/14, 14%) and one individual described anxiety as possibly masking binge eating disorder, citing that an individual with binge eating disorder may be more likely to seek treatment for their anxiety than for their binge eating (1/14, 7%). One participant described anxiety as making it more difficult for patients to maintain engagement with treatment (1/4, 7%). One participant also referenced literature finding that anxiety is not predictive of binge eating disorder (1/14, 7%). Select quotes from participants regarding the relationship between anxiety and binge eating disorder are shown below and in Table 5.

*See P38 and P75 statement in Domain 1, Theme 1*.

“*Eating disorders are as – if not more – characterized by anxiety than [by other] mood disorders. People tend to think [about] depression and [eating] disorders, but you know, rates of social anxiety are so high.” (P72)*

“*I think [anxiety – in the context of binge eating disorder] is not as well developed. It's not as prevalent in binge eating disorder samples as it seems to be in anorexia samples or bulimia samples. That's …kind of diagnostic. …Do I think that binge eating disordered individuals experience anxiety? Rumination? Worry? Yeah. So, I think [anxiety is] relevant as a phenomenon. I don't think it's as compelling in binge eating disorder as it is in the other eating disorder, as a diagnostic entity.” (P33)*

#### Theme 2: Possible mechanisms linking anxiety to binge eating disorder

Most of the 11 participants who described anxiety as being a common comorbidity with binge eating disorder (11/14, 79%, as reported in Section Theme 1: Nature of relationship between anxiety and binge eating disorder) simply acknowledged the common comorbidity but did not speculate why the comorbidity might exist. However, four of the 11 participants *did* describe possible mechanisms linking anxiety to binge eating disorder (4/14, 29%; Table 5). Two participants described anxiety as being related to possible food or micronutrient deficiencies (2/14, 14%). One participant each described anxiety as being increased by weight gain or by fears of gaining weight, being around certain foods, or related to interpersonal experiences (1/14, 7%). One participant described anxiety as being related to serotonin levels (1/14, 7%). One participant described a causal mechanism by which individuals eat to cope with- or soothe anxiety (1/14, 7%).

*See Participant 37 statement in Domain 1, Theme 2*.

“*[There are] anxiety disorders, and then [there are] anxiety … mechanisms, and then [there are] also anxiety processes within the context of the eating disorder: [e.g.,] fear of weight gain, fear [of being] around a certain food, what that's going to do. … I think in a lot of this population there are heightened interpersonal fears related to … rejection, exclusion. There's super cool research out of England that found a sensitivity to social rank there, there's just a lot around threat sensitivity we need to understand better.” (P72)*

“*The emotions go haywire when serotonin levels [get] low, so … when somebody tries to clean carbohydrate out of their diet, they're already in trouble.” (P84)*

#### Theme 3: Social anxiety disorder

Of the 11 anxiety disorders listed in the anxiety disorder section of the DSM-5 (1), social anxiety disorder was specifically spontaneously identified by three participants as a relevant comorbidity in binge eating disorder (3/14, 21%; Table 5) and was therefore identified as an independent theme here (3.3.3). Two of the participants referenced research on the role of social threat (2/14, 14%) and suggested social anxiety disorder is “the most common additional mental health problem for people with an eating disorder,” (1/14, 7%).

“*[Depending] on what literature you look at, some literature… would say the most common additional mental health problem for people with an eating disorder is social anxiety disorder. That's often though, clinical studies, which are based [more] around bulimia nervosa, but definitely social anxiety can get caught up with eating. [e.g., someone with an eating disorder doesn't] want to socialize, because so much of our socialization is around food and eating, so [s/he avoids socializing] because of that, but [one can] also permanently have social anxiety disorder as well.” (P93)*

“*As a field … we neglect social anxiety disorder because we tend to think it's just about weight and shape, self-consciousness, I think we under-diagnose this. … we need to be looking specifically at Social Anxiety Disorder and I think based on Janet Treasurer's work, we're going to end up seeing that there's links in …sensitivity to social threat, … the extent to which that's causal, secondary to the eating disorder … understanding where anxieties sort of intersect and [understanding the] neurocognitive process …especially around threat sensitivity… is going to be really helpful.” (P72)*

### Domain 3: Attention deficit disorder and attention deficit hyperactive disorder

Thirteen participants described attention deficit disorder (ADD) or attention deficit hyperactive disorder (ADHD) as relevant to binge eating disorder pathology (13/14, 93%; Table 6).

**Table 6:** Participant statements relating domain 3, “ADD/ADHD” (93%).

**Theme 1) Comorbidity**	**7 (50%)**
1) Frequently comorbid	6 (43%)
Referenced empirical support	1 (7%)
2) Comorbidity studies less clear	1 (7%)
**Theme 2) Nature of the relationship between ADD/ADHD and BED**	**7 (50%)**
1) Mechanisms underlying ADD/ADHD could be relevant to BED^a^	6 (43%)
2) Cites work related to sensory input/autism	2 (14%)
3) BED as a way to manage untreated ADD/ADHD	1 (7%)
4) Childhood ADHD may be a risk factor for adult BED	1 (7%)
5) High anxiety with food preoccupation can feel like ADD	1 (7%)
6) Possible subgroup/phenotype of BED and ADD/ADHD	1 (7%)
**Theme 3) Notes stimulant use in BED treatment (e.g., Lisdexamfetamine)**	**7 (50%)**
1) Impact of ADD/ADHD stimulant medications on appetite	2 (14%)
Not helpful	1 (7%)
Neutral	1 (7%)
**Theme 4) Relevance**	**5 (36%)**
1) Emerging research suggests potential relevance	4 (7%)
2) Some overlap but not significant enough to be relevant	1 (7%)
**Theme 5) Case findings**	**4 (29%)**
1) See a lot of untreated ADD/ADHD in BED	3 (21%)
2) Helps a lot of patients if they can get diagnosed with ADD/ADHD (when appropriate)	3 (21%)
3) Case findings historically overlooked^b^	2 (14%)
**Additional participant statements related to domain 3, theme 2, “nature of the relationship between ADD/ADHD and BED”**	
“*[From] a sensory perspective generally, …the emerging literature and in sensation that's done – particularly in the world of autism – around **sensory input**, that's starting to come over a little bit into the eating disorder world ……neurobiologically, it seems to me – based on my reading of that very early literature – that many people struggle with binge eating disorder and bulimia. So those binge-containing illnesses have a **drive for sensation, to seek sensation**, that may [be] hardwired … to drive …people …to drive fast and … take risks and ... to do adventure and to meet new people… [there may be a subset of individuals with binge eating disorder who are] **seeking sensation** and **lots of sensation feels good to them**. …Does that fit under an ADHD frame? I don't know. Does it fit under just a variation in **sensory wiring**? That's how I think about it. **It certainly seems to play out in the trait-based literature so far that people who struggle with bulimia and binge eating … tend to be a little bit more impulsive and dysregulated**. Is that ADD/ADHD? Is that just a sensory wiring that makes them a little different than people who tend to struggle with anorexia who are a little bit more risk avoidant and a little more rule constrained? I don't know.” (P60)*	
“*I think a lot of what we've done is dismiss **attentional issues** related to eating disorder specifics or anxiety-based. I think we're wrong. I think a lot of people actually probably meet full criteria.” (P72)*	
“***I see a lot of ADHD and binge eating disorder**. …there's a lot of **trauma with ADHD** also, because you see a lot of people that have … an **underperforming in their life** because they have poor executive functioning, so they are not able to do things – you know, strong start poor finish – and planning is a very difficult thing. In many people with a co-occurring mental health [illness] like ADHD, depression, …social anxiety, …whatever, many people have difficulty engaging in routines.” (P7)*	

Results expressed as *n* (%). Percentages: *n*/14 times 100.

a e.g., problems with attention, impulsivity, reward responsivity, and task initiation and completion.

b e.g., due to discrepancy between higher case findings of ADD/ADHD in boys and higher treatment rates for BED in women.

ADD, attention deficit disorder; ADHD, attention deficit hyperactive disorder; BED, binge eating disorder.

#### Theme 1: Comorbidity (50%)

Of the 13 participants who described ADD/ADHD as being *relevant* to binge eating disorder pathology, seven participants (7/14, 50%) also addressed the frequency of comorbidity between ADD/ADHD and binge eating disorder (Table 6). Six of these seven participants (6/14, 43%) described frequent comorbidity between ADD/ADHD and binge eating disorder, four referencing research findings (4/14, 29%). One participant expressed a view that comorbidity studies are unclear (1/14, 7%).

*See P75 statements in domain 1 (depression), theme 1*.

“*Frequently, frequently comorbid.” (P53)*

“*The comorbidity studies …are a little less clear. ADHD, in a diagnostic sense, doesn't seem to be something that … is highly prominent, like mood disorders, in binge eating.” (P33)*

“*Certainly, there are some statistical overlaps. My impression is that there's not a prominent diagnostic comorbidity.” (P46)*

#### Theme 2: Nature of the relationship

Seven participants addressed the possible nature of the relationship between ADD/ADHD and binge eating disorder pathology (7/14, 50%; Table 6). Six of these highlighted relevant mechanisms that underly ADD/ADHD and binge eating disorder (e.g., problems with attention, impulsivity, reward responsivity, and task initiation and completion) (6/14, 43%). Two cited work around sensory input and autism as being relevant (2/14, 14%). One participant each expressed views that: (a) binge eating may be used to manage untreated ADD/ADHD; (b) childhood ADHD may pose a risk factor for adult binge eating disorder; (c) a possible subgroup of individuals with binge eating disorder may exist who also meet diagnostic criteria for ADD/ADHD in a relevant way; and (d) that high levels of anxiety paired with food preoccupation can feel like ADD for some individuals with binge eating (1/14, 7% each).

“*The neural cognitive processes – whether it's sequential behavior, whether it's filtering out background noises, whether it's really struggling with sort of initiating and implementing behavior – I think we're going to end up seeing overlaps.” (P72)*

“*It's possible [that] separate from the diagnostic entity of ADHD, there could be attentional or impulsivity problems that people with binge eating disorder experience. I don't know that we need to invoke ADHD as a diagnostic entity to explain [the overlap]. You could look at inhibitory control, you could look at attention bias, you could look at a lot of things, so I'm not big on just jumping into [saying], ‘oh, they must have ADHD,'.” (P33)*

“*Probably resonating with [the relevance and prevalence of ADD/ADHD as a comorbidity] is that concept of impulsivity and acting without thinking, and that people with an eating disorder … most people's attention deficit disorder may have that cognitive challenge in terms of moderating impulsivity, moderating behaviors, so they act before thinking and [are] not able to interrupt that in the same way as people with more cognitive control over their impulsive thoughts and drivers [can]. So, yes, I think … you can see how [there] can be commonalities...” (P93)*

#### Theme 3: Pharmacologic management

Seven participants discussed the use of stimulant medications (e.g., Lisdexamfetamine) to treat binge eating disorder as being relevant to a possible relationship between ADD/ADHD and binge eating disorder (7/14, 50%; Table 6). Two participants discussed the impact of ADD/ADHD stimulant medications on appetite (2/14, 14%); one participant viewed the impact as “not helpful” and one participant expressed neutral views on the impact (7% each). Subthemes related to participant views on the use of stimulant medications to treat binge eating disorder will be addressed in a separate forthcoming manuscript.

“*Since Vyvanse works for both [ADD/ADHD and binge eating disorder], there should be some biological … overlap.” (P53)*

“*I think what we sort of learned as a field a while ago – probably 60 years ago now – is that stimulant medications are relatively effective as weight loss agents but they're also relatively dangerous as weight loss agents, and we don't think they're a very good idea. So, the idea of using stimulants to treat [binge eating disorder] is a different question that has to be considered differently but you can't forget that we have extensive experience with weight-related disorders with stimulants, and that that experience is not very good.” (P5)*

“*I think there is certainly some understanding in terms of … [the ability of stimulant medications in] modulating [the] drivers for the binge eating behavior, and perhaps how the stimulant is working, in terms of helping people with binge eating disorder reduce the binge eating. …it does [suggest] that there is …overlap with the use of stimulants in attention deficit disorder that is helping people get a little bit more …control [of their] behaviors, so that's probably acting at a biological … neurocognitive level.” (P93)*

“*I do get [clients who are] put on …Topamax even, and I find it not helpful because then they're not having an appetite. So, I do have clients that don't have eating disorders that are put on stimulants and it's really a hinderance because they're still having issues with their ADD and ADHD, and then now they have no appetite, and they don't want to eat. So … big issue there.” (P37)*

#### Theme 4: Relevance

Five participant statements addressed the relevance of ADD/ADHD to binge eating disorder (5/14, 36%; Table 6). Four of these participants expressed views that more research is emerging suggesting the potential relevance of ADD/ADHD to binge eating disorder pathology (4/14, 29%). One participant acknowledged some overlap may exist between ADD/ADHD and binge eating disorder but “not enough to be relevant,” (1/14, 7%).

“*I think [ADD and ADHD are] particularly relevant and important [in the context of binge eating disorder]. I think we're finding more and more research suggesting that that is a pathway that is … very relevant comorbidly, and particularly in kids – to understand people who might be at risk for binge eating disorder.” (P19)*

#### Theme 5: Disparities in evaluation

Four participant statements related to case findings of ADD/ADHD in adult binge eating disorder (4/14, 29%; Table 6). Three participants reported seeing a lot of untreated ADD/ADHD in patients with binge eating disorder (3/14, 21%). Three participants reported benefits to the patient if they can get diagnosed with ADD/ADHD when applicable (3/14, 21%). Two participants stated ADD/ADHD case findings and prevalence have been historically overlooked due to misconceptions about adult ADD/ADHD (e.g., that it's only present if diagnosed in childhood) and due to higher case findings of ADD/ADHD and binge eating disorder treatment-seekers in boys and adult females, respectively (2/14, 14%).

“*A lot of our patients are really helped if you can get that piece [of existing ADD/ADHD] diagnosed. So, there's that piece.” (P72)*

“*I think that, in general across eating disorders, [Attention Deficit Disorder or Attention Deficit Hyperactive Disorder is] something that probably is more common than we… have tended to notice in the past. I think part of that is [because] there aren't as many chances for case findings [of binge eating disorder in men and] that means that they're mostly women in the treatment programs [and]… historically ADHD or …ADD case finding has been much more effective in young boys than in young girls. …if a young boy is having behavioral aberrations in elementary school, people think about ADHD very quickly. We see a fair number of women in our treatment programs who have ADHD where … they weren't in trouble all the time and if they did at least adequately academically nobody thought about it.” (P5)*

### Domain 4: Substance-related and addictive disorders

Eleven participants described substance-related and addictive disorders (SRADs) as being relevant to binge eating disorder (11/14, 79%; Table 7). Nine participants (9/14, 64%) expressed views that SRADs are common or relevant comorbidities or co-occurring conditions with binge eating disorder. Three participants expressed views that individuals with binge eating disorder may experience co-occurring eating disorders and SRADs in such a way that one is worsened when another improves (3/14, 21%). One participant described a co-occurrence of tobacco use and binge eating disorder, in which individuals may smoke cigarettes to curb appetite in attempt to manage binge eating (1/14, 7%). One individual expressed an understanding that high prevalence rates of familial comorbidity exist (1/14, 7%). One individual emphasized the importance of screening for SRADs (1/14, 7%). Views on SRAD comorbidity that related to views on food/eating addiction are addressed in a separate manuscript from this study.

“*I think there's a bit of data that folks with binge eating disorder have an increased frequency of substance use disorders. … I don't think it's extreme, but I think there was some. I think it's true for all eating disorders. (P46)*

“*Co-occurring addiction: we see a lot; that's very common.” (P5)*

*See P38 and P75 statement in Domain 1 (Depression), Theme 1*.

“*I've heard from plenty of patients [with comorbid substance-related and addictive disorders that] they feel like it's whack-a-mole, right? They're like, ‘Okay, now my eating disorder is better. Nope, now my addictions are worse. Nope, now, my addiction is better [but] my eating disorder is worse.' … I think there's a lot of progress around that. And again, especially as we're understanding reward mechanisms, but you know, we need to think about that piece.” (P72)*

**Table 7:** Participant statements relating to domain 4, “substance-related addictive disorders.”

1) SRADs are relevant to BED	11 (79%)
2) SRADs are common or relevant co-occurring conditions	9 (64%)
3) When SRADs and BED co-occur, one is worsened when another improves	3 (21%)
4) High prevalence rates of familial comorbidity	1 (7%)
5) Importance of screening for SRADs	1 (7%)
6) Tobacco use to curb appetite	1 (7%)
**Additional participant statements related to subtheme S1.4, “Substance-related addictive disorders”**	
“*We definitely see a **high familial comorbidity** … a genetic link between a familial history of addiction and propensity toward binge-type eating disorders. We also see a **subset of individuals who show both of those comorbidities**. … In general, what I found in my research is actually more of a … **sequential** [relationship]: … people who've previously had a substance use disorder are prone to developing a binge eating disorder [and vice versa]. And then particularly with **cigarettes** is where I see it couldn't be more co-occurring. … people will be using cigarettes as an attempt to… reduce their eating behavior… **I don't think I've seen a lot of research on the flip side, where people who have binge eating disorder got better and developed a substance use disorder**, I don't think anybody's ever studied that to my knowledge.” (P19)*	

Results expressed as *n* (%). Percentages: *n*/14 times 100. BED, binge eating disorder; SRAD, substance-related addictive disorder.

### Domain 5: Obsessive-compulsive disorder

#### Theme 1: Comorbidity

Ten participants commented on the prevalence between obsessive compulsive disorder (OCD) and binge eating disorder (10/14, 71%; Table 8). Seven participants (7/14, 50%) expressed views that as a comorbidity, obsessive-compulsive disorder (OCD) is more prevalent and/or relevant in anorexia nervosa and/or bulimia nervosa than in binge eating disorder. Six participants (6/14, 43%) expressed views that OCD is less or not very prevalent/relevant in binge eating disorder (one of those participants cited literature). Two participants (2/14, 14%) stated they were not aware of any research on comorbidities between OCD and binge eating disorder. One participant expressed a view that a high prevalence exists between OCD and binge eating disorder (1/14, 7%). Another participant expressed a view that the prevalence is probably overlooked (1/14, 7%).

“*You see [OCD co-occurring in binge eating disorder] occasionally, it's much more common in people who have [anorexia nervosa] than it is in people who have [binge eating disorder], in my experience, and I think that probably fits pretty well with published literature as well.” (P5)*

“*I think of OCD as less relevant to [binge eating disorder], relative to anorexia nervosa.” (P19)*

“*I think it's potentially less important, at least [based on] the comorbidity studies that I look at” (P33)*

**Table 8:** Participant statements relating to domain 5, “OCD.”

**Theme 1) Prevalence**	**10 (71%)**
OCD is more prevalent/relevant in AN/BN vs. BED	7 (50%)
OCD is less/not very prevalent in BED	6 (43%)
Not aware of research on comorbidity	2 (14%)
High comorbidity prevalence of BED and OCD	1 (7%)
Comorbidity prevalence likely overlooked	1 (7%)
**Theme 2) Mechanistic overlap**	**11 (79%)**
OCD and BED may share overlapping aspects	6 (43%)
Some overlap attributed to nutrition (research cited)	1 (7%)
Not much overlap between OCD and BED	4 (29%)
Not aware of research on overlap	2 (14%)
**Theme 3) Clinical considerations**	**2 (14%)**
Possible OCD phenotype across all EDs	1 (7%)
Need to screen for OCD in BED	1 (7%)
**Additional participant statements related to domain 5, theme 2, “mechanistic overlap”**	
“*I think [the possible co-existence of obessionality and compulsivity around food or eating in binge eating disorder is an] interesting … aspect of any eating disorder because even in binge eating disorder, you're going to find people with **malnutrition**. Malnutrition doesn't discriminate. [And] we know that malnutrition can affect **food preoccupation**. …the Ancel Keys study [was] a perfect example of that, where we see food preoccupation. Of course, [in the Ancel Keys study], they were starving, but with poor nutrition, we see preoccupation with food, so I think that it's always interesting.” (P37) (31)*	

Results expressed as *n* (%). Percentages: *n*/14 times 100.

AN, anorexia nervosa; BED, binge eating disorder; BN, bulimia nervosa; ED, eating disorder; OCD, obsessive compulsive disorder.

#### Theme 2: Mechanistic overlap

Six participants (6/14, 43%) expressed views that OCD and binge eating disorder may share overlapping aspects (e.g., obsessionally and compulsivity) that may be relevant to understanding binge eating disorder pathology (Table 8). One of these participants attributed some of the overlap to malnutrition, citing findings that malnutrition can cause obsession and compulsion around food and eating (1/14, 7%). Four participants expressed views that there is not much overlap between OCD and binge eating disorder, and that the two entities are “two different things,” (4/14, 29%).

“*What you see a lot in people who have [anorexia nervosa] is really diagnosable OCD. If you think about* ***obsessionality or compulsivity*** *as something that can not only be part of OCD, but rather as* ***personality traits****, or, you know*, ***commonly seen behaviors or*** ***tendencies****, well, that cuts pretty broadly across the eating disorders. I don't think it's more so true in those with [binge eating disorder], but you see it across the board.” (P5)*

“*…the* ***compulsivity system is certainly* …*shared*** *in these sorts of disorders, but my understanding is that OCD prevalence is more highly related to anorexia than [to] binge eating disorder.” (P19)*

“*Phenotypically there's rough correlates. But I mean, in eating disorders, there were OCD spectrum models 20 years ago, which – when they went back and did the* ***family history*** ***studies*** *to see if in fact OCD was elevated in family members of eating disordered people – they were negative.” (P33)*

#### Theme 3: Clinical considerations

One participant suggested the existence of a phenotypic subgroup that exists across all eating disorders in which individuals express high levels of obsession, compulsion, and perfectionism (1/14, 7%; Table 8). Another expressed a view that OCD should be screened for (1/14, 7%).

*See P93 statements in Domain 3 (ADD/ADHD), Theme 2*.

### Domain 6: Post-traumatic stress disorder

Nine participants commented on the relationship between post-traumatic stress disorder (PTSD) and binge eating disorder (9/14, 64%; Table 9). Six participants (6/14, 43%) described observing associations between PTSD and binge eating disorder, either clinically/anecdotally (“we see it,”) or in the literature. Four participants described PTSD as an important risk factor for binge eating disorder (4/14, 29%). Two participants stated that any trauma is “bad for the brain,” (2/14, 14%). One participant expressed a view that it is important to consider not just “full-blown PTSD,” but also sub- or undiagnosed PTSD, linking this statement to the important of assessing effect size in research (1/14, 7%). One participant described PTSD as exacerbating binge eating disorder and reported binge eating as being used as a way of coping with PTSD (1/14, 7%). One participant expressed a view that PTSD can make binge eating disorder treatment more difficult to tolerate as an outpatient (1/14, 7%).

“***We see lots of people who have PTSD*.** *And I don't think we have data to prove this is the case, but I think one of the things that having PTSD may do is [that] it may* ***make it harder to tolerate the distress*** ***that comes with doing treatment as an outpatient*.** *Any level of treatment brings distress, and, in some ways, I think part of providing the treatment is finding strategies that allow somebody to tolerate doing what we need to do treatment-wise. … so that means that* ***in higher-intensity treatment settings, we see lots of people with*** ***PTSD.” (P5)***

“*I think for …* ***the relative minority of people who have had those kinds of traumatic*** ***experiences****, especially if they have* ***post-traumatic stress symptoms****, following those traumatic experiences, I think it's* ***potentially extremely relevant to their eating disorder*,** ***psychopathology****.” (P33)*

“*[PTSD is a] huge component [of adult binge eating disorder], I think, because again, it's* ***just a way of coping****. But I do I think* ***trauma is something I see a lot*** *and …* ***it may take time to uncover itself****, but … PTSD [is often present] as well.” (P37)*

**Table 9:** Participant statements relating to domain 6, “PTSD.”

**Theme 1) Relationship**	**10 (71%)**
An association between PTSD and BED is observed (clinically or in the literature)	6 (43%)
PTSD as an important risk factor for BED	4 (29%)
Any trauma is bad for the brain	2 (14%)
Important to distinguish PTSD from sub- or undiagnosed PTSD (important to assess effect size in research)	1 (7%)
PTSD can exacerbate binge eating/binge eating as a way of coping with PTSD	1 (7%)
PTSD can make BED treatment harder to tolerate as an outpatient	1 (7%)
**Additional participant statements related to domain 6, “PTSD”**	
“*[PTSD is] a risk factor for any psychiatric problem.” (P53)*	
“*…the way it typically goes [that] early life trauma or trauma precedes eating disorders, often, and in other words, it's not as often the other way around.” (P38)*	
“*We definitely see the **association of binge eating … with PTSD** …they do appear to be more comorbid with each other. I would say that … my only question is… **a lot of people experienced trauma and don't develop full blown PTSD and … I've never looked at the difference in effect size between … full blown PTSD and … the experience of trauma itself**. You're definitely going to have …a bigger basket … if it's just about the experience of deprivation and trauma, and …I could imagine that … more people are going to be at risk.” (19)*	

Results expressed as *n* (%). Percentages: *n*/14 times 100.

BED, binge eating disorder; PTSD, post-traumatic stress disorder.

## Discussion

### Novelty and innovation

To the authors' knowledge, our study is among the first to synthesize expert opinion on mental health factors pertaining to adult binge eating disorder pathology. Further, our study identifies important connections and possible underlying mechanisms that link a variety of mental health issues to adult binge eating disorder. These connections highlight the need for clinical screening of adult binge eating disorder among individuals with other Axis-I mental health disorders (and vice versa) and the need for updated information about current prevalence rates of mental health issues among adults with binge eating disorder (with- and without a formal diagnosis).

### Relationship of findings to existing literature

Table 1 displays participant recognition of possible comorbidities between binge eating disorder and several other Axis-I mental health disorders alongside lifetime associations for various DSM-4/CIDI mental health disorders, as observed in WHO-WMH study, the WHO-NCR, NESARC-III, SQCDS, and SNEDQRPRS (2, 4, 13–15). Depression and anxiety seem to be the most commonly recognized mental health comorbidities among experts participating in this study (Domains 1 and 2). This recognition aligns with the NESARC-III and SNEDQRPR surveys, which found major depressive disorder to have highest sample prevalence rates among participants with binge eating disorder (32.3–65.5%) (15) and with the WHO-NCR and SQCDS studies, which found anxiety disorders to have the highest population prevalence (55.5–65.1%) (2, 13). However, WHO-WMH found ADD/ADHD to have the highest population prevalence rates (9.3%, noting that some countries were excluded from the population as they did not assess for this disorder). Again, it must be noted these surveys were conducted between 2001 and 2013 in populations of individuals who already had a formal DSM binge eating disorder diagnosis. Both binge eating disorder and OCD have undergone diagnostic reconfiguration with the release of the DSM-5, following collection of these data (1, 17). Moreover, our understanding of binge eating disorder prevalence and correlates has evolved significantly as a field, as have prevalence and correlates rates themselves. Thus, new information on mental health correlates and prevalence rates among individuals with binge eating disorder is warranted, as is information on the mechanisms that underlie the relationships between mental health- and binge eating disorder psychopathology in adults. It should also be acknowledged that a higher prevalence of Axis I disorders among those with binge eating disorder does not necessarily translate to a higher prevalence of binge eating disorder among those with Axis I disorders. Thus, information on prevalence rates of binge eating disorder among Axis I disorders is also warranted.

### Clinical implications

Our results call to light the need for updated information on mental health correlates and prevalence rates in adult binge eating disorder. Currently, the most up to date information available was obtained between 2001 and 2013 (Table 1) (2, 4, 13–15). These data were collected or extracted prior to the release of the DSM-5 in 2013, when binge eating disorder became its own autonomous eating disorder diagnosis and OCD was reclassified as no longer being an anxiety disorder (1). Moreover, mental health prevalence rates continue to increase at large, particularly in the wake of the Coronavirus 19 pandemic, and more recent studies suggest 93.4–96.8% of individuals who meet DSM criteria for binge eating disorder never receive a formal diagnosis (18, 19). Thus, updated information on mental health correlates of adult binge eating disorder is warranted.

Fortunately, some such information is beginning to emerge. For example, a prospective observational longitudinal panel study in Brazil explored the relationship between anxiety, depression, and binge eating symptoms in 155 patients (124 women) with obesity recruited from a university-based bariatric center assessed at 3, 6, and 9 months after Brazil's nationwide lockdown in March 2020 (29). The study's path analysis found that anxiety had an effect on binge eating at 3- and 9-months post-lockdown (ß = 0.39 and 0.26, respectively) and binge eating affected depressive symptoms at 9 months post-lockdown (ß = 0.22) but was the most unstable symptom over time. These findings illustrate the nature of the relationships between binge eating disorder, depression, and anxiety, and underscore the importance of evaluating and treating psychiatric symptoms of binge eating disorder, anxiety, and depression in association with one another and over time.

Our findings similarly underscore the importance of equal and adequate screening for mental health comorbidities among individuals with binge eating and for screening for binge eating disorder among individuals with other mental health problems. Expert opinion suggests that on the one hand many individuals with binge eating disorder may have comorbid mental health disorders that go untreated or under-treated and possibly exacerbate binge eating. On the hand, many individuals with binge eating disorder and comorbid mental health disorders may be more open to seeking treatment for another mental health disorder, rather than for binge eating disorder. This may be due in large part to internal and external stigmatization of binge eating disorder, as addressed in Bray et al. (27).

The importance of adequate screening for binge eating disorder cannot be under stated. Current findings suggest that 67.3% of individuals who meet DSM criteria for binge eating disorder do not perceived the need for treatment (18); 93.4–96.8% never receive a formal diagnosis (18, 19); and 56.4–86.8% never receive or pursue treatment (2, 18). This is especially true for minority and marginalized populations that are historically overlooked in the field (27). Thus, equal and adequate screening for binge eating disorder as well as other mental health disorders offers a first step toward helping those who suffer find the help they need and improving mental health outcomes for individuals with binge eating disorder at large.

### Study limitations and strengths

The study's sample size is appropriate for a mixed-methods analysis of this nature but limits the ability to generalize the data's themes and conclusions to the field of binge eating disorder researchers and clinicians at large. Thus, findings from this study are not necessarily generalizable. Further, the themes identified in this work have not been verified by the experts themselves. Additionally, this study did not collect data on expert opinion on several important and often comorbid mental health disorders, including schizophrenia or schizoaffective disorder and personality disorders, as well as other mental health disorders whose comorbidities with binge eating disorder are less known (e.g., autism spectrum disorder and Asperger's syndrome). The parent study did collect data on expert perspectives about how other forms of trauma (excluding PTSD) relates to binge eating disorder; however, these data are published elsewhere (27).

Another study limitation is that one of the study's four possible eligibility criteria for researchers (NIH R01 grant funding; see Table 1) presents a bias for including participants from the U.S. There were 3 other eligibility criteria researchers could meet to be included that were not dependent on any nationality and the final study sample does include participants from the UK, AU, and CA as well as from the US. Nevertheless, it would have been optimal to including criteria for funding form other federal agencies. Additionally, this study collected demographic data on sex assigned at birth but not gender. This oversight is important because gender is demographically relevant whereas sex assigned at birth is not relevant to this study question. Further, asking for sex assigned at birth follows an old convention that fails to account for equity and diversity inclusion.

This study utilizes several methodological strengths to counterbalance the limitations identified above. First, this study's systematic inclusion criteria (Table 2) provides strong population representation of experts who drive the field. This includes researchers with the greatest funding and publication output (recently and historically) and clinicians with high clinical and academic affiliations as well as those most likely to be accessed by individuals with binge eating disorder themselves (e.g., most commonly identified through a Google search or through popular press books on binge eating disorder). Second, the study sample includes a well-rounded balance of binge eating disorder experts, including researchers, medical doctors, licensed therapists, licensed/registered dieticians, intuitive eating specialists, healthcare administrators, and public health advocates (Table 3). Although one of the four possible inclusion criteria for researchers involves NIH funding, this criterion was not required (researchers were required to meet one of the four possible criteria). Thus, while this criterion presents a bias for inclusion of academics within the U.S., the study sample does include a balanced geographic representation, with individuals across the U.S. as well as in the UK, AU, and CA.

### Conclusions

Overall, adult binge eating disorder is linked to a wide range of mental health domains in the literature (30). These links are generally recognized by most experts in the field, and it is important for clinicians to screen for binge eating disorder among individuals with other Axis-I mental health issues (and vice versa). Research on underlying mechanisms that link various mood disorders to binge eating disorder is recommended by the experts. Updated global and national surveys that collect information on mental health correlates and prevalence rates among individuals with binge eating disorder is also warranted.

## Data availability statement

The original contributions presented in the study are included in the article/supplementary material, further inquiries can be directed to the corresponding author.

## Ethics statement

The studies involving human participants were reviewed and approved by the National University of Natural Medicine (NUNM) IRB (# HZ12120). Written informed consent for participation was not required for this study in accordance with the national legislation and the institutional requirements.

## Author contributions

BB and HZ: conceptualization, methodology, formal analysis, investigation, and project administration. BB: resources, data curation, and writing—original draft preparation. BB, CB, HZ, and RB: writing—review and editing. HZ and RB: supervision. All authors have read and agreed to the published version of the manuscript and agree to be accountable for the content of the work.

## Funding

This research was funded by NCCIH, Grant Number: 5R90AT008924-07 and by NCCIH, Grant Number: 1K24AT011568-01.

## Conflict of interest

The authors declare that the research was conducted in the absence of any commercial or financial relationships that could be construed as a potential conflict of interest.

## Publisher's note

All claims expressed in this article are solely those of the authors and do not necessarily represent those of their affiliated organizations, or those of the publisher, the editors and the reviewers. Any product that may be evaluated in this article, or claim that may be made by its manufacturer, is not guaranteed or endorsed by the publisher.
